# Ectasie cornéenne suite à une kératomycose

**DOI:** 10.11604/pamj.2014.17.229.3470

**Published:** 2014-03-27

**Authors:** Hakima Elouarradi, Lalla Ouafae Cherkaoui

**Affiliations:** 1 Université Mohammed V Souissi, Service d'Ophtalmologie A de l'hôpital des spécialités, Centre Hospitalier Universitaire, Rabat, Maroc

**Keywords:** Ectasie cornéenne, kératomycose, infection, Corneal ectasia, keratomycosis, infection

## Image en medicine

Les kératomycoses représentent une cause souvent grave, d'infection cornéenne. Leur survenue est favorisée par l'usage intensif des corticoïdes, et des lentilles de contact. Les champignons responsables sont souvent opportunistes et envahissent des cornées pathologiques. Les kératomycoses peuvent également survenir sur des cornées normales après traumatisme souvent par un végétal. Les infections à Fusarium et Aspergillus sont les plus fréquentes. Le mauvais pronostic de ces infections est dû à la virulence des champignons qui infectent souvent des cornées déjà pathologiques mais aussi à des retards diagnostiques et thérapeutiques (coût élevé des nouveaux traitements). Nous rapportons l'observation d'un patient âgé de 16 ans, sans antécédents particuliers, hospitalisé pour un abcès de cornée de l'oeil gauche suite à un traumatisme par épine végétale 1 semaine avant, les prélèvements du grattage cornéen étaient négatifs. Le diagnostic d'abcès mycosique posé devant l'interrogatoire, le terrain et l'aspect clinique très évocateur. L’évolution était marquée par l'apparition 2 mois plus tard, d'une ectasie cornéenne avec amincissement important, une distension limbique et une chambre antérieure profonde à l'examen à la lampe à fente. L'acuité visuelle était limitée à une perception lumineuse.

**Figure 1:**
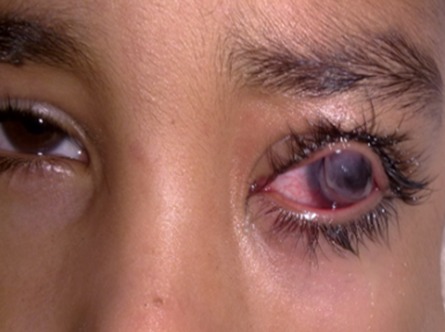
Ectasie cornéenne et amincissement central important post infectieux

